# Novel Insights of Oligometastases and Oligo-Recurrence and Review of the Literature

**DOI:** 10.1155/2012/261096

**Published:** 2012-08-22

**Authors:** Yuzuru Niibe, Joe Y. Chang

**Affiliations:** ^1^Department of Radiology and Radiation Oncology, School of Medicine, Kitasato University, 1-15-1 Kitasato, Minami-ku, Kanagawa Sagamihara 252-0374, Japan; ^2^Department of Radiation Oncology, The University of Texas, MD Anderson Cancer Center, Houston, TX 77030, USA

## Abstract

Oligometastases and oligo-recurrence are among the most important notions of metastatic and recurrent cancer. The concept of oligometastases is related to the notion that cancer patients with 1–5 metastatic or recurrent lesions that could be treated by local therapy achieve long-term survival or cure, while the concept of oligo-recurrence is related to the notion that cancer patients with 1–5 metastatic or recurrent lesions that could be treated by local therapy have controlled primary lesions. Achievement of long-term survival or cure in patients with oligometastases and oligo-recurrence is cancer and organ specific. These facts rely on the seed and soil theory and multiple steps of cancer progression. Oligo-recurrence is considered to have a better prognosis than oligometastases. In patients with oligometastases and oligo-recurrence, the oligometastases and oligo-recurrence are sometimes cured with only local therapy, which is an example of the abscopal effect, previously described in relation to cure of lesions outside of the field of radiation therapy without systemic therapy. Oligometastases and oligo-recurrence can now be cured by less invasive local treatment methods combined with systemic therapy. The mechanisms of oligometastases and oligo-recurrence, as well as novel insights into these important concepts, are presented in this paper.

## 1. Introduction

 Oligometastases and oligo-recurrence are among the most important notions of metastatic and recurrent cancer [1, 2]. These notions are now widely accepted by oncologists, and many reports of oligometastases and oligo-recurrence have been published. The concept of oligometastases is related to the notion that cancer patients with 1–5 metastatic or recurrent lesions that could be treated by local therapy achieve long-term survival or cure. However, the status of the primary lesion of these cancer patients has no restrictions, though patients with active primary lesions have a worse prognosis than patients with controlled primary lesions. Niibe et al. showed that the most important prognostic factor of oligometastases was the status of the primary lesion [3]. On the other hand, the concept of oligo-recurrence is related to the notion that cancer patients with 1–5 metastatic or recurrent lesions that could be treated by local therapy have controlled primary lesions [2]. Then, the biggest prognostic factor for oligometastases is overcome in oligo-recurrence. This is a very important point in oligo-recurrence. This notion has been proposed by Niibe et al. [2]. Another important point in oligo-recurrence is that the oligometastases are metachronous. Synchronous oligometastases have an active primary lesion. However, metachronous oligometastases almost always have a controlled primary lesion except for concomitant primary and distant recurrence (sites: 1–5).

 Furthermore, achievement of long-term survival or cure in patients with oligometastases and oligo-recurrence is cancer- and organ-specific. These facts rely on the seed and soil theory and multiple steps of cancer progression [4, 5]. The seed and soil theory remains an accepted notion in modern biology and oncology [6–8]. The cancer cells' interactions with host organs are very complicated and specific at the level of gene mutation, gene expression, molecular expression, MET-EMT cross-talk, and so on [6–10]. The multiple steps of cancer progression indicate that cancer cells in the primary lesion are not monoclonal and have a different metastatic potential [5]. Recently, cancer stem cells have been reported to play an important role in cancer progression and metastasis [11, 12].

 In this paper, mechanisms of oligometastases and oligo-recurrence are discussed through a review of the literature and our experience, and novel insights into these mechanisms are presented.

## 2. Mechanisms of Oligometastases

In this paper, oligometastases are defined as the state in which patients have 1–5 metastatic or recurrent lesions with active primary lesions. This definition prevents confusing oligometastases with oligo-recurrence. Another way of considering this status is sync-oligometastases, in which cancer patients have 1–5 synchronous metastases with active primary lesions, excluding metachronous metastases.

 Metastasis has been recently reported to arise from cancer stem cells [11, 12]. Primary tumor sites consist of various metastasis-potential cancer cells. Of these, cancer stem cells have metastasis potential, which is produced by cancer gene mutations. This means that sync-oligometastases cancer patients already have gene mutations in primary cancerous lesions [5, 11, 12]. Moreover, tumor-host cross-talk in gene mutations, gene expression, molecular expression, and MET-EMT interactions lead to organ-specific metastases [5, 6, 9, 10]. In nonsmall cell lung cancer (NSCLC), oligometastases often arise in patients who have brain-only or adrenal-only metastases [3, 13, 14]. In small cell lung cancer (SCLC), oligometastases often arise in patients who have brain-only metastases [15]. In uterine cervical cancer, oligometastases often arise in patients who have para-aortic lymph node-only metastases [16], while in colorectal cancer, oligometastases often manifest as liver-only metastases [17, 18]. These sync-oligometastases could be cured by local therapy combined with systemic therapy. In this situation, local therapy should treat both metastatic lesions and primary lesions to pursue cure or long-term survival.

 Recently, Lussier et al. indicated that oligometastases enhanced by MicroRNA-200c lead to polymetastases after local radiation therapy [19]. This is a new finding related to cancer multistep progression. If MicroRNA-200c has not been enhanced in oligometastases, polymetastases do not occur. However, this has limitations, in that oligometastases occur in an organ-specific manner. This is explained by the above-mentioned modern seed and soil theory.

## 3. Mechanisms of Oligo-Recurrence

Oligo-recurrence is the state in which cancer patients have metachronous metastases after curative therapy for primary lesions. At recurrence, the cancer patients have no relapse of the primary lesions. This is very important with respect to local therapy. With local therapy it is relatively easy to treat 1–5 metastases and recurrences in one organ. However, primary lesion treatment is usually difficult with local therapy and includes radiation therapy, surgery, and radiofrequency ablative therapy, because primary lesion recurrence often involves regional lymph node metastases or invasion to adjacent organs. Furthermore, oligo-recurrence is the state of metachronous oligometastases. This is why we consider oligo-recurrence to have a better prognosis than sync-oligometastases.

 Oligo-recurrence is also cancer and organ specific. The seed and soil theory is adapted in oligo-recurrence. In NSCLC, oligo-recurrence often arises with brain-only recurrences [3]. In uterine cervical cancer, oligo-recurrence often involves para-aortic lymph node-only recurrences [20–22]. In colorectal cancer, oligo-recurrence often involves liver- and lung-only recurrences [17, 23].

At the time of treatment for the primary lesion, oligo-recurrent cancer patients might have one to several micrometastases. These micrometastases remain dormant for a period. These then grow and can be detected by computed tomography, magnetic resonance imaging, positron emission tomography, and increasing tumor marker levels. This state is oligo-recurrence, with one to several gross recurrences. Interleukin has been reported to play a key role in the growth of micrometastases [24]; it is the switch that results in progression of micrometastases.

## 4. Relationship between the Abscopal Effect and Oligometastases and Oligo-Recurrence

The abscopal effect is defined as tumor outside of the irradiation field disappearing without systemic therapy when the radiation therapy target tumor is irradiated. This is a rare phenomenon. We have reported the abscopal effect in uterine cervical cancer [25] and hepatocellular carcinoma [26]. Other reports have documented the abscopal effect in malignant melanoma [27], malignant lymphoma [28], and others.

**Figure 1:**
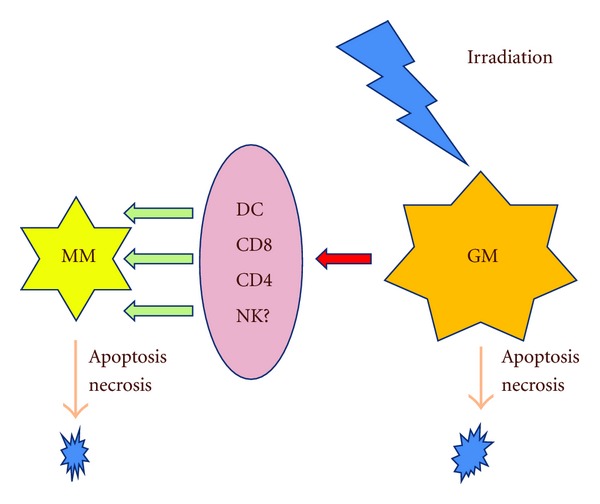
Relationship between the abscopal effect and disappearance of micrometastases. Abbreviations: GM: gross metastasis, DC: dendritic cell, NK: natural killer cell, MM: micrometastases.

 In patients with oligometastases and oligo-recurrence, radiation oncologists, oncologic surgeons, and interventional oncologists have sometimes found that oligometastases and oligo-recurrence have been cured with only local therapy. These patients are considered to have micrometastases. However, gross metastases and recurrent lesions treated by radiation therapy, surgery, and radiofrequency ablative therapy lead to cure. This phenomenon is considered to be the abscopal effect. The abscopal effect is reported to occur with surgery, as well radiation therapy [29]. The abscopal effect could diminish micrometastases (Figure 1), so that oligometastases and oligo-recurrence treated only by local therapy may sometimes be cured.

## 5. Relationship between Systemic Therapy and Oligometastases and Oligo-Recurrence

Punglia et al. reported that the survival benefit of local therapy increased as systemic therapy improved [30]. Niibe et al. reported that the survival benefit of local therapy increased dramatically as systemic therapy improved, indicating their original figure designating the sigmoid-curve relationship between increasing survival benefit of local therapy and improving systemic therapy [2]. This figure is very important because it revises the previous figure to a sigmoid-curve. Recently, systemic therapy has been improving, and the importance of local therapy, especially in cases with minimal invasiveness, is increasing dramatically. Stereotactic body radiation therapy (SBRT), intensity modulated radiation therapy (IMRT), proton therapy, heavy ion therapy, radiofrequency ablative therapy (RFA), video-assisted partial surgery, and robotic surgery are less invasive than therapies of a decade ago. These methods now apply to sync-oligometastases and oligo-recurrence combined with systemic therapy, including molecular-targeted therapy. With these, patients can benefit from improved outcomes with less invasive and treatments that are more likely to be successful for sync-oligometastases and oligo-recurrence.

## 6. Clinical Outcomes

### 6.1. Oligometastases and Oligo-Recurrence in the Lungs

Oligometastases and oligo-recurrence in the lungs treated by surgery were reported to achieve good outcomes in the 1990s, in a large population study [31] (Table 1). The International Registry of Lung Metastases (IRLM) reported a 5-year overall survival rate of 36% among 5206 patients with lung metastases treated by surgery. This report suggested that origin of the germ cell tumor was favorable survival. In 2009, oligometastases of colorectal cancer in the lungs treated by surgery were also found to achieve favorable survival [32]. Three hundred and seventy-eight patients underwent pulmonary resection for colorectal cancer metastases with curative intent, and a 3-year overall survival rate of 78% was achieved. This indicated that oligometastases of colorectal cancer are favorable candidates for curative-intent therapy.

Since the 2000s, stereotactic body radiation therapy has rapidly spread as medical physics improved. Stereotactic body radiation therapy has been revealed to be equivalent to surgery in tumor ablation [33]. In oligometastases of the lungs, Okunieff et al. reported a local control rate of 94% (median follow-up: 18.7 months) and a 2-year progression-free survival rate of 16% in patients treated with stereotactic body radiation therapy using mainly 50 Gy/5 fr [34]. Among them, cancer of breast or lung origin had better prognosis than those of other origins. Norihisa et al. reported a local control rate of 90% and an overall survival rate of 84.3% in patients after a 2-year followup using primarily 48–60 Gy/4-5 fr [35], and suggested that no differences existed between different tumor origins. One prospective study also reported that the local control rate was 96% and overall survival rate was 39% in patients after a 2-year followup [36]. In oligo-recurrence of the lungs, Takahashi et al. reported a local control rate of 87% and an overall survival rate of 65% in patients after a 2-year followup using 20–56 Gy/1–7 fr, and suggested that those of colorectal cancer origin had a better prognosis than others [37]. Inoue et al. reported an overall survival rate of 54% in patients after a 5-year followup using 40–48 Gy/4 fr, and suggested that disease-free interval (DFI) ≥36 was a significantly favorable prognostic factor [38].

### 6.2. Oligometastases and Oligo-Recurrence in the Liver

The most frequent liver metastases occur in colorectal cancer. However, colorectal cancer patients with liver metastases resected by surgery achieve favorable survival. The 5-year overall survival rate is about 40%–50% [39, 40] (Table 2). Furthermore, Adam et al. reported that initially unresectable colorectal liver metastasis could be cured by surgery after downsizing chemotherapy [41]. The cure rate was reported to be as high as 19%. Bismuth also reported that initially unresectable colorectal liver metastases could achieve a 5-year overall survival rate of 40% [42].

SBRT is also applied to liver metastases, as lung metastases can be curable by SBRT. van der Pool et al. reported that the 2-year local control and 2-year survival rates in colorectal cancer patients with liver metastases (i.e., mostly colorectal cancer) were 74% and 83%, respectively, after treatment with SBRT using mainly 37.5 Gy/3 fr [43]. Romero et al. conducted a prospective trial to treat liver metastases with SBRT using mainly 37.5 Gy/3 fr [44], and reported that the 2-year local control and 2-year overall survival rates were 86% and 62%, respectively. Rusthoven et al. conducted a prospective trial on liver metastases treated with SBRT using 36–60 Gy/3 fr [45], and found that the 2-year local control and survival rates were 92% and 30%, respectively, which indicated that favorable prognostic factors were the origins of colorectal cancer, breast cancer, and renal cell cancer. 

## 7. Conclusions

The mechanisms of oligometastases and oligo-recurrence were reviewed, and novel insights are presented. Sync-oligometastases and oligo-recurrence can now be cured by less invasive local treatment methods combined with systemic therapy.

## Figures and Tables

**Table 1:** Outcomes of oligometastases and oligo-recurrence in the lung.

Author	Year	Study design	Treatment method	Local control (%)	Overall survival (%)
IRLM [31]	1997	retrospective	surgery	—	36 (5 years)
Onaitis et al. [32]	2009	retrospective	surgery	—	78 (3 years)
Okunieff et al. [34]	2006	retrospective	SBRT	94 (median followup: 18.7 mo.)	16 (PFS)
Norihisa et al. [35]	2008	retrospective	SBRT	90 (2 years)	84.3 (2 years)
Rusthoven et al. [36]	2009	prospective	SBRT	96 (2 years)	39 (2 years)
Takahashi et al. [37]	2012	retrospective	SRS, SBRT	87 (2 years)	65 (2 years)
Inoue et al. [38]	2012	retrospective	SBRT	—	54 (5 years)

Abbreviations: SBRT: stereotactic body radiation therapy; SRS: stereotactic radiosurgery; mo.: months.

**Table 2:** Outcomes of oligometastases and oligo-recurrence in the liver.

Author	Year	Study design	Treatment method	Local control (%)	Overall survival (%)
Choti et al. [39]	2002	retrospective	surgery	—	40 (5 years)
Pawlik et al. [40]	2005	retrospective	surgery	—	58 (5 years)
Adam et al. [41]	2009	retrospective	chemotherapy → surgery	—	33 (5 years)
Bismuth et al. [42]	1996	retrospective	chemotherapy → surgery	—	40 (5 years)
van der Pool et al. [43]	2010	retrospective	SBRT	74 (2 years)	83 (5 years)
Romero et al. [44]	2006	prospective	SBRT	86 (2 years)	62 (5 years)
Rusthoven et al. [45]	2009	prospective	SBRT	92 (2 years)	30 (5 years)

Abbreviations: SBRT: stereotactic body radiation therapy.
